# Severe Pediatric Polytrauma Complicated by Stroke After Fall From Swamp Buggy

**DOI:** 10.7759/cureus.87067

**Published:** 2025-06-30

**Authors:** Morgan C Uebelacker, Avram Rago, Joseph Fahmy, Alexandria Farish

**Affiliations:** 1 Emergency Medicine, University of South Florida Morsani College of Medicine, Tampa, USA; 2 Pediatrics, University of South Florida Morsani College of Medicine, Tampa, USA; 3 Pediatric Emergency Medicine, Tampa General Hospital, Tampa, USA

**Keywords:** blunt cerebrovascular injury, emergency medicine, pediatric emergency medicine, pediatric stroke, pediatric trauma, swamp buggy accident, traumatic carotid dissection, traumatic cervical spine injury

## Abstract

Blunt cerebrovascular injuries (BCVIs) are rare but potentially severe complications of blunt trauma and can lead to acute ischemic stroke. Diagnosis requires imaging but necessitates more cautious consideration in pediatrics due to radiation concerns. Management options span observation, anticoagulation, and various endovascular interventions, but decisions are complicated by limited literature and the presence of other traumatic injuries. Further collaborative research is needed to provide clarity when caring for pediatric patients with BCVI to guide diagnosis and management.

A four-year-old male with no significant past medical history presented to the emergency department as a trauma activation secondary to injuries sustained after falling from a moving “swamp buggy” recreational vehicle. He was found to have severe craniofacial and cervical spine injuries including open bilateral mandibular fractures, C2-C3 distraction injury with associated spinal cord injury, and blunt left common carotid injury subsequently complicated by a left hemispheric stroke with right hemiplegia. Management challenges arose due to the complexity of injuries, with decisions regarding management of initial injuries and subsequent stroke treatment being particularly intricate. The patient underwent various interventions, including closed reduction of spinal fractures, halo vest placement, mandibular fracture repair and ultimately open internal fixation of spinal fractures via spinal fusion.

Despite the severity of injuries, the patient had a positive outcome, underscoring the importance of multidisciplinary collaboration in trauma care. This is an uncommon injury presentation in a pediatric patient and this case was highlighted to demonstrate the diagnostic and treatment challenges regarding blunt cerebrovascular injuries in pediatric patients. Further research is needed to guide physicians caring for pediatric patients with blunt cerebrovascular injuries and the sequela of such injuries.

## Introduction

As a leading cause of mortality and morbidity in children, traumatic injuries account for more than 8.7 million emergency department visits by children each year [1]. This case demonstrates a unique constellation of traumatic injuries in a pediatric patient sustained by falling from a moving “swamp buggy” and the sequela including open bilateral mandibular fractures, C2-C3 distraction injury with associated spinal cord injury, blunt left common carotid injury and subsequently a left hemispheric stroke. Blunt cerebrovascular injuries (BCVIs) are rare, occurring in only approximately 3% of adult blunt trauma patients [2] and <1% of pediatric blunt trauma patients [3]. Acute ischemic stroke is a feared complication of BCVIs that represents a significant degree of morbidity and mortality. This is an uncommon injury presentation in a pediatric patient and this case highlights the need for continued research surrounding diagnosis and management of pediatric blunt cerebrovascular injuries and their sequela. This case was previously presented as a poster at the Florida College of Emergency Physicians 2024 Symposium by the Sea meeting on July 27, 2024, in Miami, Florida.

## Case presentation

A 20kg four-year-old male with no significant past medical history presented to the emergency department as a trauma activation after falling from a moving vehicle. He was an unrestrained and unhelmeted passenger riding on an elevated back seat of an open-top “swamp buggy” off-road vehicle when he struck the top of a garage door as the driver was parking and fell off the vehicle, approximately 6 to 10 feet. Bystanders called 911 and a police officer was the first to arrive on scene. The patient was reportedly unresponsive and pulseless prompting the use of an automated external defibrillator to deliver one defibrillation with subsequent return of spontaneous circulation; initial rhythm is unknown. It is unknown if CPR was performed and for how long.

When local ground Emergency Medical Services (EMS) arrived on scene, the patient had a pulse but was apneic and unresponsive with a Glasgow Coma Scale (GCS) score of 3. He was subsequently intubated in the field with a 4.5 uncuffed endotracheal tube (ETT) after facilitation with 40mg ketamine IV and 6mg etomidate IV. The ground EMS crew called for critical care air transport to minimize out-of-hospital time and expedite trauma team evaluation. In addition to the medications used to facilitate intubation, he also received 25mcg fentanyl IV, 100cc 3% saline IV and 400mg levetiracetam IV while en route to the hospital with the flight crew. 

Pediatric surgery was present upon patients' arrival to the emergency department (ED) and assisted the pediatric emergency medicine team with management. Upon arrival to the ED, ETT placement was confirmed via the presence of bilateral breath sounds and waveform capnography. Heart rate was 101 beats per minute, blood pressure 126/99 mmHg, oxygen saturation was 100% and respiratory rate was 28 on mechanical ventilator, and his temperature was 97.6° Fahrenheit. GCS was 7T (M4V1E2) and cervical collar was already in place. He had obvious facial and neck trauma with a full-thickness laceration from the left nasolabial fold extending into the left cheek, with exposed mandible noted in the mouth. There was a non-expanding hematoma to the left neck without palpable thrill or pulsatility however his trachea was deviated to the right. His pupils were equal and reactive to light bilaterally and he was withdrawing his bilateral upper extremities to pain and moving his bilateral lower extremities spontaneously. The remainder of the primary and secondary survey did not demonstrate any other clinically significant findings. Initial and secondary Focused Assessment with Sonography for Trauma (FAST) exams were performed after the primary and secondary surveys and were unrevealing. Initial chest and pelvis X-rays (XR) in the trauma bay demonstrated the ETT in trachea without any other obvious acute traumatic injuries. Continuous dexmedetomidine and fentanyl infusions were initiated at 0.2mcg/kg/hr and 1mcg/kg/hr for sedation and analgesia respectively and an emergent femoral central line was placed for vascular access. He underwent stat trauma imaging including computed tomography (CT) head, CT cervical spine, CT facial bones, CT angiogram (CTA) brain, CTA neck, and CT chest, abdomen and pelvis with contrast. 

**Figure 1:**
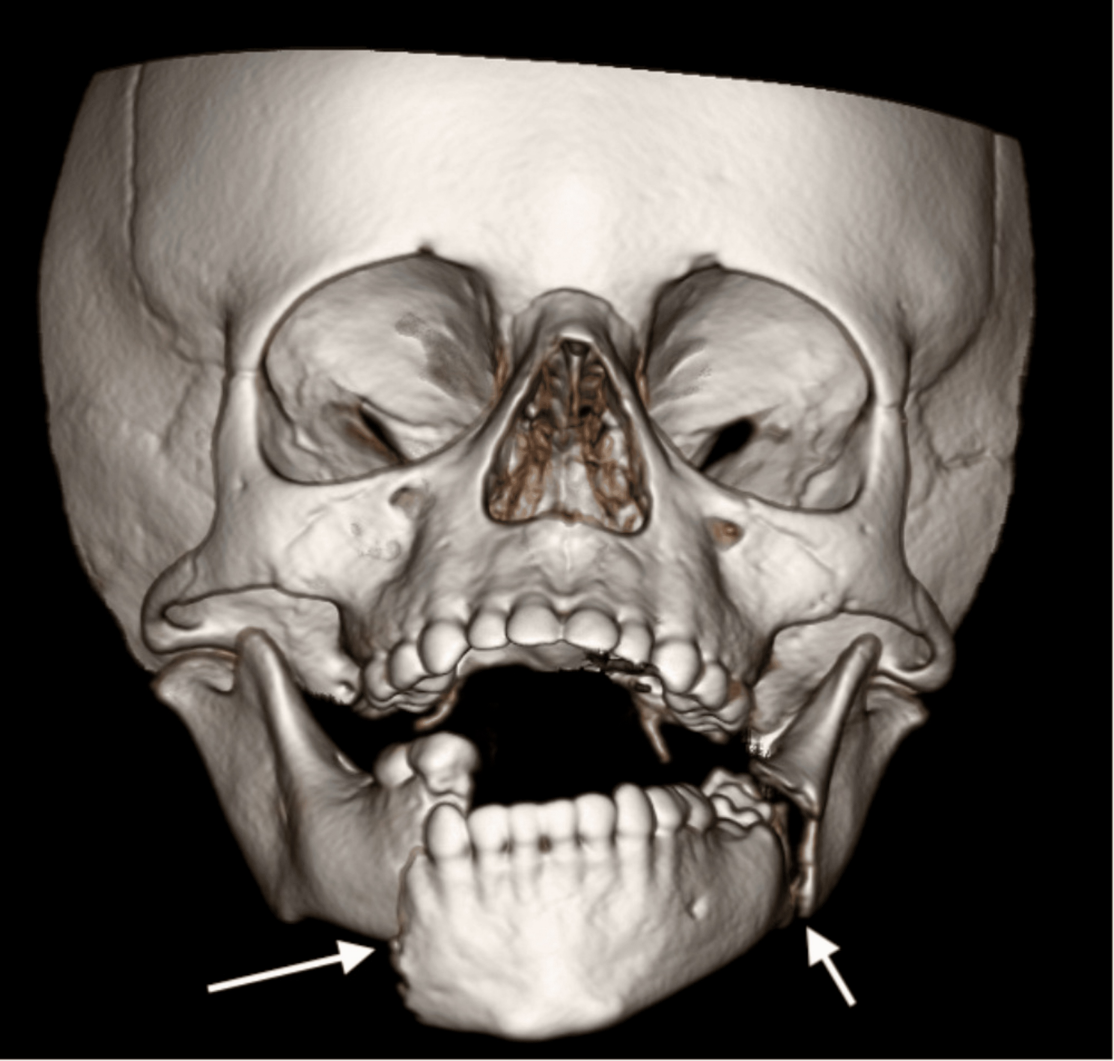
3D reconstruction of CT facial bones demonstrating acute bilateral mandibular fractures through the body of the right hemimandible and at the angle of the left hemimandible

**Figure 2:**
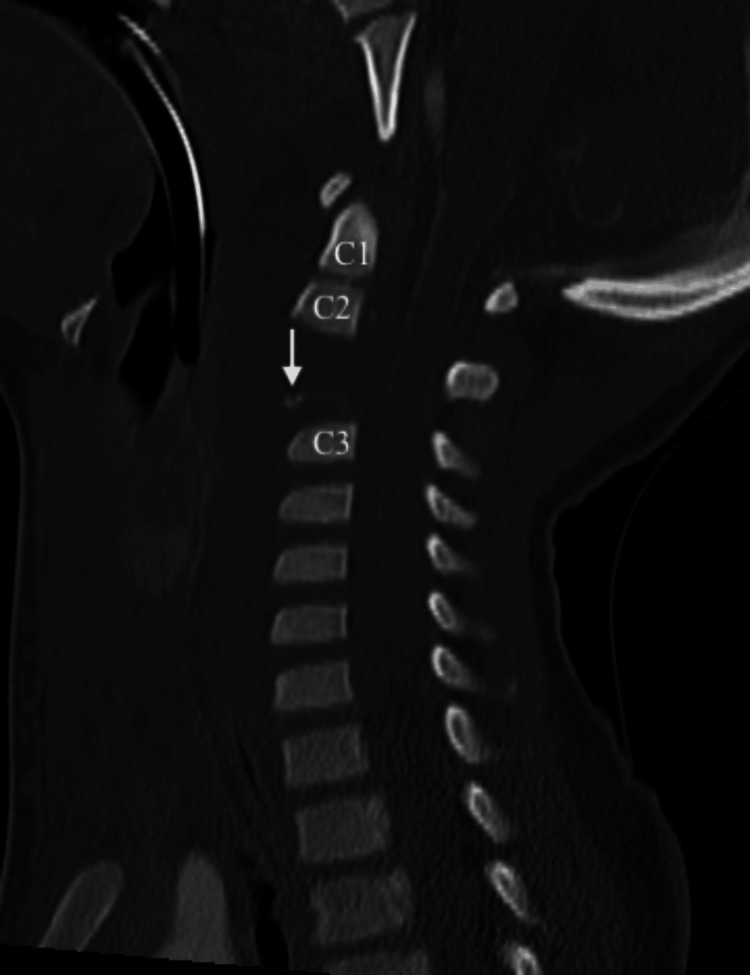
Sagittal view of CT C-spine demonstrating C2-C3 distraction injury with avulsion fracture of C2 with 1.1 cm distraction of the base of C2

**Figure 3:**
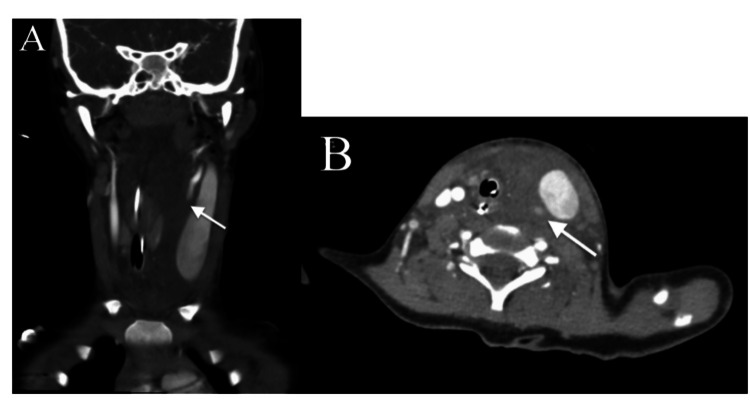
CT angiogram neck from hospital day 0 demonstrating loss of opacification of the left carotid artery with surrounding hematoma and resultant left jugular venous distension and tracheal deviation to the right in coronal (A) and axial (B) planes.

CT head and CTA brain demonstrated no acute intracranial abnormalities. CT facial bones revealed acute bilateral mandibular fractures with significant displacement (Figure 1). CT C-spine demonstrated a C2-C3 distraction injury with 1.1cm distraction of the base of C2 with associated ligamentous injuries, and a long segment epidural hematoma (Figure 2). CTA neck was concerning for a left common carotid transection versus dissection with a large surrounding hematoma extending into the prevertebral and retropharyngeal spaces causing tracheal deviation (Figure 3). There were also findings concerning for left internal jugular vein injury with jugular venous distension and luminal irregularities. CT chest, abdomen and pelvis did not demonstrate any other traumatic injuries.

**Figure 4:**
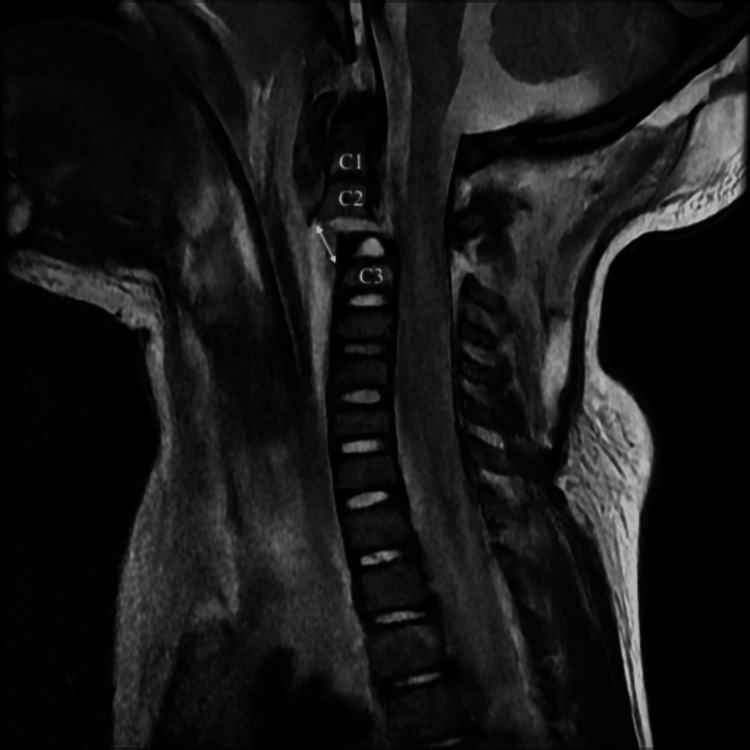
Sagittal T2 C-spine magnetic resonance imaging (MRI) demonstrating avulsion fracture at the base of C2 and malalignment of cervical spine.

**Figure 5:**
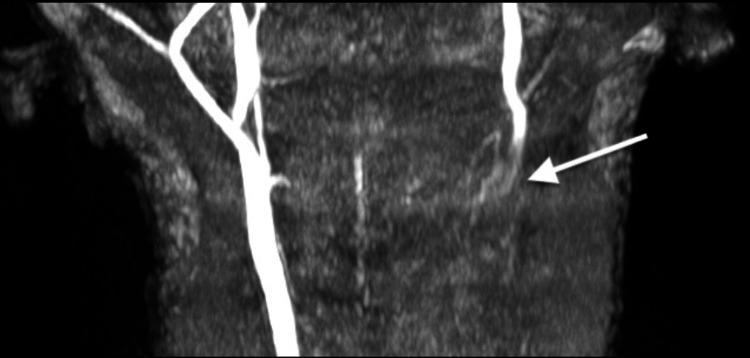
Coronal view of magnetic resonance angiogram (MRA) demonstrating blunt injury of left common carotid with loss of flow signal in the entirety of the visualized left common carotid.

Neurosurgery, vascular surgery, endovascular neurosurgery, otolaryngology (ENT) and pediatric critical care were consulted for further management. He subsequently underwent magnetic resonance imaging (MRI) of the brain, C-spine and MR angiogram (MRA) of neck while still in the ED. Brain MRI was normal. MRI of the C-spine re-demonstrated the C2-C3 distraction injury with an acute avulsion fracture of the base of C2 body with 1.1cm distraction, anterior subluxation of the base of C2, subluxation of the left C2-C3 facet joint, as well as significant associated ligamentous injuries. Also noted on MRI was spinal cord edema from C2-C3, a thin ventral epidural hematoma from C5-T2, a thin dorsal epidural hematoma from T3-T4, and prevertebral and left carotid space hematomas (Figure 4). MRA of the neck re-demonstrated the blunt vascular injury of the left common carotid artery extending to the origin of the cervical internal carotid (Figure 5). These findings were felt to represent a thrombus in the true lumen versus a dissection with thrombus in the false lumen occluding the true lumen; however, given the large surrounding hematoma, transection could not be ruled out. 

After a multidisciplinary discussion between vascular surgery, endovascular neurosurgery and pediatric surgery, it was felt that CTA and MRA neck imaging findings were reflective of a grade IV carotid artery dissection and thrombosis with surrounding hematoma rather than complete transection. It was also felt that any endovascular or surgical interventions would be high risk for embolization and stroke given the extent of injury and the teams opted for conservative management at that time with watchful waiting and hourly neurologic checks. Anti-thrombotic therapy with aspirin was recommended once he was cleared by neurosurgery. Due to these vascular injuries, neurosurgery did not feel the patient was stable for prone positioning and open surgical repair of his cervical spine injuries and they opted to proceed with emergent closed reduction and stabilization of the fracture via halo device placement with plans for open internal fixation at a later date.

Prior to going to the operating room with neurosurgery, the patient became hypotensive to 60/37 mmHg refractory to normal saline fluid bolus at 20cc/kg, so he was started on a continuous epinephrine infusion at 0.1-0.3mcg/kg/min to maintain mean arterial pressures greater than 65mmHg. He was taken from the ED to the operating room (OR) emergently with neurosurgery for closed reduction of unstable C-spine injury via halo vest placement and was post-operatively admitted to the pediatric intensive care unit (PICU) under the care of the pediatric surgery service as well as pediatric critical care. After arrival at the PICU post-operatively, ENT repaired the complex facial laceration at bedside and pediatric oral maxillofacial surgery was consulted for further management of mandibular injuries. He received intravenous cefazolin 25mg/kg every eight hours for infection prophylaxis until open mandibular fractures could be repaired pending clinical stability and repair of cervical spine injury.

**Figure 6:**
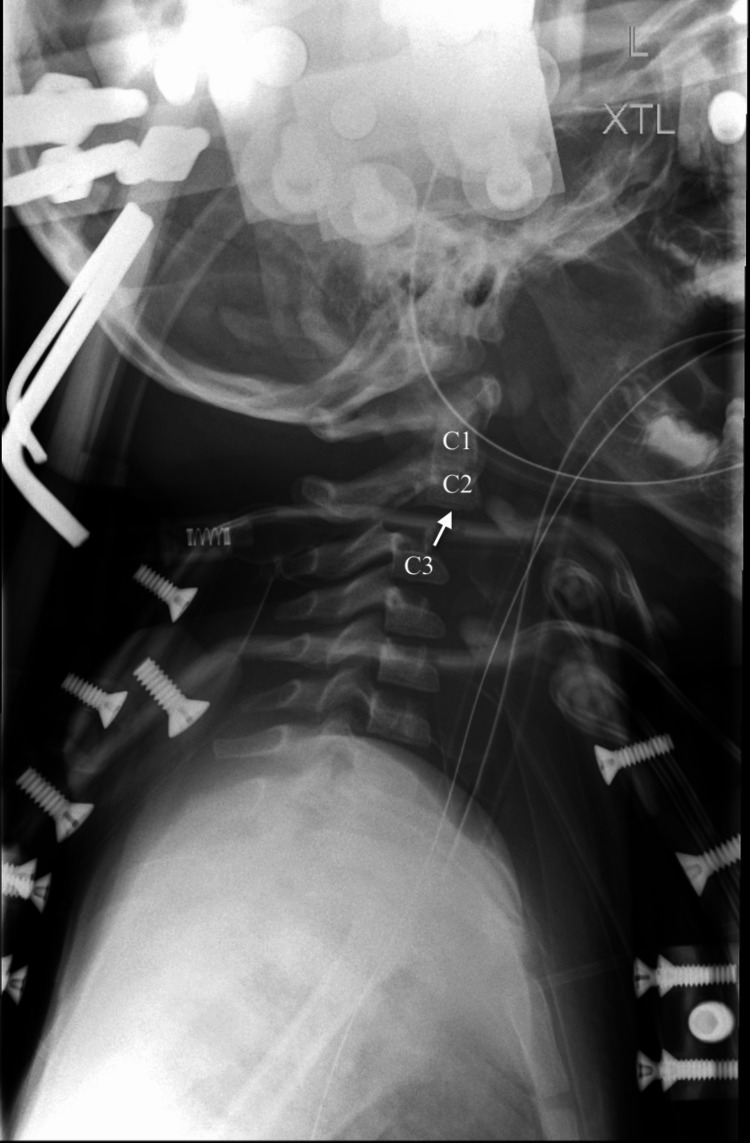
Lateral C-spine X-ray demonstrating unsatisfactory reduction with distraction and anterior subluxation of C2 relative to C3

**Figure 7:**
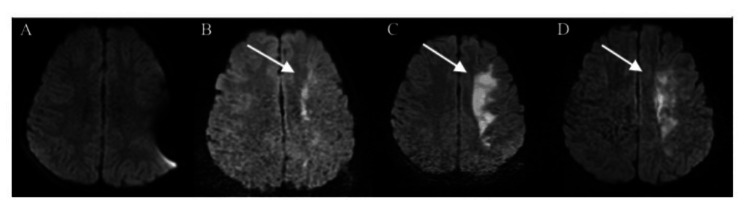
Diffusion-weighted brain magnetic resonance images (MRIs) demonstrating an evolving hyperintensity in the left cerebral hemisphere. Initial MRI from hospital day 0 (A) did not show any signs of ischemia however subsequent MRIs on hospital day 1 (B), hospital day 3 (C) and hospital day 6 (D) demonstrated evolving cytotoxic edema from the ischemic infarct.

On hospital day 1, he underwent repeat C-spine XR to evaluate alignment following closed reduction and halo placement and unsatisfactory reduction was noted (Figure 6). At this point, neurosurgery hoped to take him back to the OR with initial plans for open internal fixation and posterior fusion. However, prior to going to the OR, it was noted by nursing staff that the patient was no longer withdrawing from pain on his right upper or lower extremities during a routine neurologic check. Given underlying cerebrovascular injuries, there was concern for acute stroke and the hospital stroke protocol was activated, prompting immediate evaluation by stroke neurology at bedside as well as diagnostic imaging. He underwent stat CT head, CTA head and neck as well as MRI brain and MRI C-spine to assess progression of spinal cord injury. Brain MRI demonstrated interval development of an acute left cerebral hemisphere stroke in a watershed distribution (Figure 7). C-spine MRI re-demonstrated the C2-3 fracture-dislocation with persistent anterior subluxation of C2 relative to C3 causing high-grade canal stenosis with cord edema. Despite the acute ischemic event, he was not a candidate for thrombolytic therapy given recent trauma, spinal cord injury and carotid injury. Further, he was not a candidate for endovascular treatment with thrombectomy or stenting due to underlying carotid injury given risk of further embolic phenomenon to patent intracranial vessels and high likelihood of hemorrhagic conversion if initiated on dual-antiplatelet therapy or anticoagulation. Thus, medical management of ischemic stroke was felt to be the safest option including permissive hypertension targeting a mean arterial pressure greater than 85mmHG to maintain cerebral perfusion pressure for 48 hours as well as antiplatelet therapy with aspirin 81mg once daily for seven days followed by 40mg once daily until later cleared by pediatric neurology. He was also given a levetiracetam load of 20mg/kg followed by daily levetiracetam maintenance for seizure prophylaxis at 200mg twice daily. Subsequent brain MRI 48 hours later did not show any worsening of ischemia or any signs of hemorrhagic conversion (Figure 7). 

**Figure 8:**
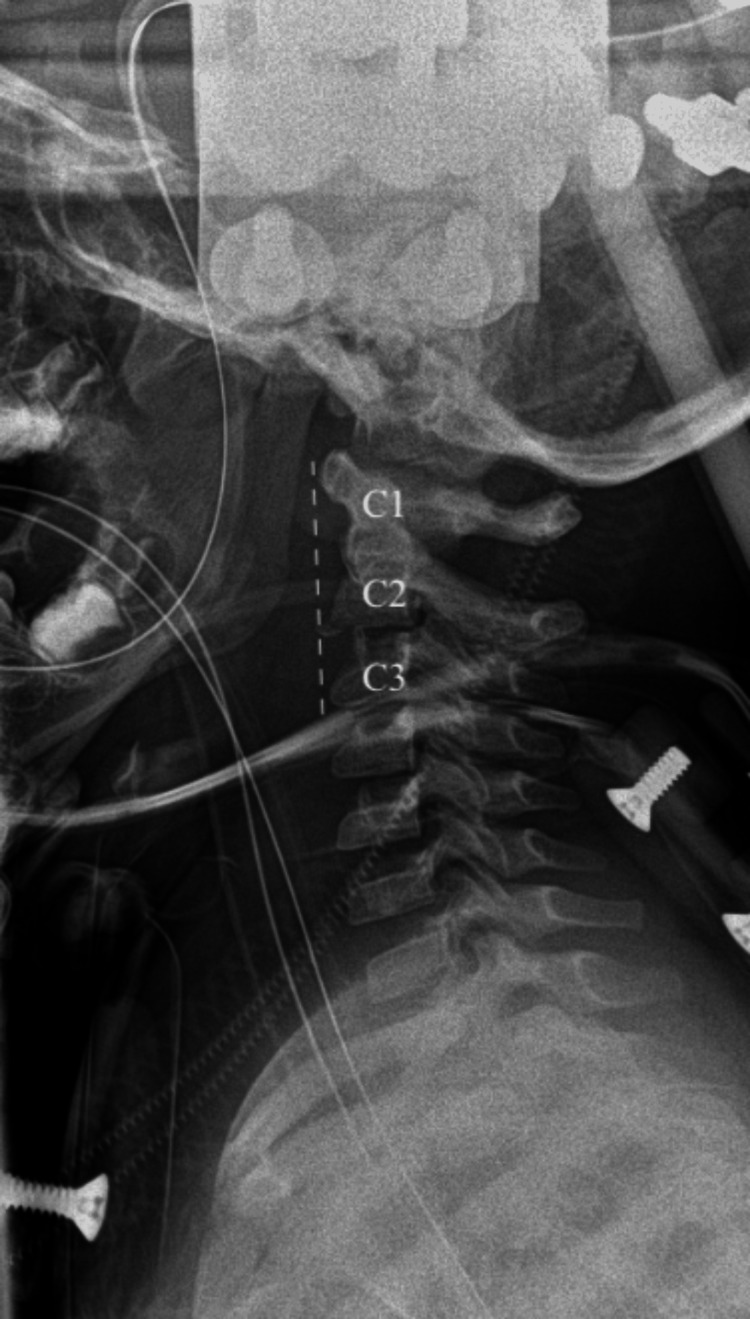
Lateral cervical spine X-ray from hospital day 1 following revision of halo device in the operating room demonstrating more satisfactory alignment of cervical spine when compared to Figure 6

Despite malalignment of C-spine fracture-dislocation noted on C-spine MRI, open internal fixation and posterior fusion was again deferred in the setting of acute stroke. However, the patient did return to the OR on hospital day 1 with neurosurgery for closed revision of the halo with adequate reduction of fracture-dislocation and better anatomic alignment on post-operative XR (Figure 8). Ultimately, he underwent posterior spinal fusion of C2-C3 on hospital day 8 and remained in the halo vest post-operatively for continued stabilization of cervical spine. 

After definitive management of cervical spine injuries was obtained via spinal fusion, the patient was deemed stable to undergo surgical repair of mandibular fracture-dislocations with the pediatric oral maxillofacial surgery (OMFS) team on hospital day 10. To obtain adequate surgical approach by the OMFS team, the oral ETT needed to be exchanged for a nasotracheal tube which was done in the OR by anesthesia via flexible fiberoptic bronchoscope. After surgical repair of mandibular fractures, he returned to the PICU and was subsequently extubated to room air without complication the same day. Given the complexity of underlying craniofacial and cervical spine injuries as well as the high likelihood of airway edema resultant from being intubated for 10 days and multiple ETT exchanges, he was considered a critical airway and remained on airway watch for the first 24 hours post-extubation. At this point he had shown significant improvement in his neurologic exam but still had residual weakness in his right upper and lower extremities.

He was transferred out of the PICU and to the pediatric floor on hospital day 13 and subsequently transferred to the care of the pediatric inpatient rehabilitation service on hospital day 18, where he continued to make significant improvements with physical therapy (PT), occupational therapy (OT) and speech therapy. As a result of his prolonged intubation, maxillofacial trauma, carotid injury and hematoma with tracheal and esophageal deviation, nasogastric tube was placed to facilitate enteral nutrition and was maintained until hospital day 15 when he was cleared for a soft diet.

**Figure 9:**
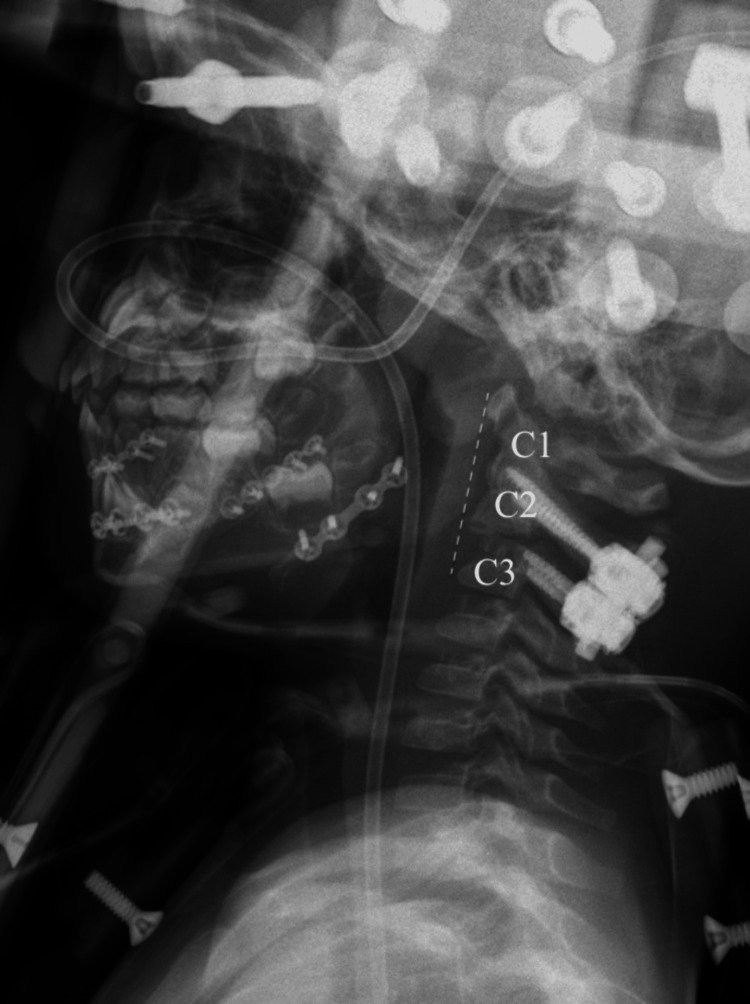
Lateral c-spine X-ray on hospital day 27 demonstrating continued satisfactory alignment of cervical spine following posterior fusion at C2-C3.

On hospital day 27, there was concern for developing infection at left frontal halo pin site so he underwent c-spine XR to evaluate alignment of c-spine and to determine if halo device could be removed. XR at that time demonstrated near anatomic alignment with stable post-operative changes from C2-C3 fusion (Figure 9); thus, the halo device was removed by neurosurgery on hospital day 29 and a cervical collar was then used to maintain stabilization until he would be cleared by neurosurgery at a later follow-up visit. He completed a seven-day course of oral cephalexin 25mg/kg every eight hours for the pin site infection. Ultimately, he was discharged from the hospital on day 39 with some minor residual right hemi body weakness but was able to ambulate independently and continued to make excellent progress with outpatient PT and OT. Per chart review from one year after initial injury, the patient had no residual neurologic deficits.

## Discussion

This case demonstrates a unique constellation of traumatic injuries in a pediatric patient sustained by falling from a moving “swamp buggy” and the sequela. This patient suffered a blunt left common carotid injury with a grade IV dissection complicated by ischemic stroke. As previously mentioned, BCVIs are rare but can lead to significant morbidity with acute ischemic stroke being a dreaded complication. The severity of these injuries can range broadly from mild endoluminal irregularities in the vessels to complete transection. Injury severity can be assessed with the Denver/Biffl scale based on imaging findings (Table 1) [4]. Higher grade carotid injuries are associated with higher risk of subsequent stroke [4,5]. With the advent of validated adult BCVI screening scores, screening CTA for blunt trauma patients has become increasingly common in recent years, especially in the adult population [3,6]. In contrast, screening criteria for pediatric patients should minimize unnecessary radiation exposure while also facilitating early, accurate diagnosis and treatment of these injuries. Attempts to apply adult screening criteria to pediatric patients have proven unreliable [3] indicating a need for a pediatric-specific screening tool. The McGovern score (Table 2) was developed specifically to screen pediatric patients for BCVIs based on previously identified risk factors such as GCS ≤ 8, focal neurologic deficits, carotid canal fracture, petrous temporal bone fracture and cerebral infarction on CT, while also taking into account the mechanism of injury (MOI) as it has been found that many pediatric patients diagnosed with BCVI were involved in high velocity motor vehicle collisions or automobile-pedestrian incidents [3,6]. A score of ≥ 3 points suggests high risk and indicates the need for angiography [3,6]. The McGovern score was recently validated in a retrospective multicenter study demonstrating a sensitivity of 80% and a negative predictive value of greater than 98% [3,6]. The McGovern score for the patient discussed in this study was a 3 based on GCS and mechanism of injury.

**Table 1:** Biffl/Denver Classification

Biffl/Denver Classification [4]	Imaging Findings	Risk of Stroke with Carotid Artery Injury
Grade I	Minimal luminal irregularity or intramural hematoma/dissection with <25% luminal narrowing	8%
Grade II	Intramural hematoma/dissection with ≥25% luminal narrowing, intraluminal thrombus, or raised intimal flap	14%
Grade III	Pseudoaneurysm	26%
Grade IV	Occlusion	50%
Grade V	Transection with free extravasation	100%

**Table 2:** McGovern Score GCS: Glasgow Coma Scale

McGovern Score [3,6]	Points
GCS ≤ 8	1
Focal neurologic deficit	2
Carotid canal fracture	2
Mechanism of injury	2
Petrous temporal bone fracture	3
Cerebral infarction on CT	3

As with the wide spectrum of injury severity, the management of these injuries also exists on a broad spectrum from observation alone to antiplatelet/anticoagulant therapy to endovascular intervention such as stenting. Existing literature regarding management of pediatric BCVI and their ischemic sequela is extremely limited and less defined than management of the same injuries in adults [4]. Additionally, the management of the ischemic sequela can be complicated by underlying traumatic injuries thus further limiting management options. Thus, further collaborative research between trauma surgery, stroke neurology and endovascular neurosurgery teams is needed to provide clarity. Thrombolysis, anticoagulation and endovascular interventions, such as thrombectomy and stenting, were felt to be too high risk in this case given high risk for bleeding, emboli and stroke provocation as well as potential worsening of underlying vascular injury. Management of BCVI in this case was complicated by underlying cervical spine trauma thus anti-platelet therapy could not be started at time of diagnosis of initial BCVI. It is unclear if this affected his outcome, given that the risk of stroke based on initial injury was already 50%. 

## Conclusions

In this case report, we discussed an uncommon injury presentation in a pediatric patient and the sequela of the initial injuries and subsequent clinical course. The positive outcome in this case, despite the severity of the initial injuries, highlights the importance of a multidisciplinary approach in caring for patients with traumatic injuries not only on their initial presentation but throughout their hospital stay as well as throughout rehabilitation. Further research is needed to guide physicians caring for pediatric patients with traumatic blunt cerebrovascular injuries and the sequela of such injuries. 
